# Identifying Liver Cancer and Its Relations with Diseases, Drugs, and Genes: A Literature-Based Approach

**DOI:** 10.1371/journal.pone.0156091

**Published:** 2016-05-19

**Authors:** Yongjun Zhu, Min Song, Erjia Yan

**Affiliations:** 1 College of Computing and Informatics, Drexel University, Philadelphia, PA, United States of America; 2 Department of Library and Information Science, Yonsei University, Seoul, Republic of Korea; University of Georgia, UNITED STATES

## Abstract

In biomedicine, scientific literature is a valuable source for knowledge discovery. Mining knowledge from textual data has become an ever important task as the volume of scientific literature is growing unprecedentedly. In this paper, we propose a framework for examining a certain disease based on existing information provided by scientific literature. Disease-related entities that include diseases, drugs, and genes are systematically extracted and analyzed using a three-level network-based approach. A paper-entity network and an entity co-occurrence network (macro-level) are explored and used to construct six entity specific networks (meso-level). Important diseases, drugs, and genes as well as salient entity relations (micro-level) are identified from these networks. Results obtained from the literature-based literature mining can serve to assist clinical applications.

## Introduction

Scientific literature is the primary source for scholars to communicate with others as well as the public. Scholars publish papers and present research outcomes in conferences to convey ideas and disseminate knowledge to the community. As online accessibility to scholarly literature is enhanced, the growth rate of scholarly literature is unprecedentedly high. A linear growth of publications has been reported for fields such as bioinformatics [1]. A concern as a result of such proliferations is the lagged consumption of scientific literature. To alleviate this tension, scholars have attempted to apply a variety of text mining techniques, such as information extraction [2], topic modeling [3], and document summarization [4], to systematically distill knowledge from large scientific literature corpora.

In biomedicine, scientific literature, primarily from PubMed [5] ―a free portal to publications and citation in Medline, has been employed in relation to text mining techniques to aid biomedical research. The focus is typically to extract relations among biomedical entities such as protein-disease associations [6], gene relations [7], gene-drug relations [8, 9, 10], gene-disease relations [11, 12], and protein-protein interactions [13, 14]. Al-Mubaid & Singh [6] applied a text mining approach to Medline abstracts to discover protein-disease association and confirmed that literature-based approach is capable of discovering associations between proteins and diseases. In the same vein, Stephens and colleagues [7] proposed a method to detect gene relations from Medline abstracts and highlighted the strength of literature-based methods that is the ability to analyze large volume of data in a limited time. Chang & Altman [8] proposed a method to extract gene-drug relations from literature and showed the effectiveness of a co-occurrence method to extract gene-drug relations in published articles (at the 78% accuracy level). Similarly, Chun and colleagues [11] proposed a system that used a co-occurrence-based machine learning algorithm to automatically extract relations between genes and relations from Medline, and emphasized the importance of gene and disease dictionaries. Temkin & Gilder [13] proposed a method that used context-free grammar to extract protein interactions from unstructured texts. They reported that the proposed method recorded a precision rate of 70% for extracting interactions among proteins, genes and small molecules (PGSM). In addition to relation identification, studies have also focused on extracting entities such as genes [15] and chemical entities [16]. Stapley & Benoit [15] extracted genes from literature by using gene co-occurrence information curated in genomic databases to improve biomedical information retrieval. Grego & Couto [16] applied a semantic similarity validation-based method to enhance the identification of chemical entities. They showed that the method can be used as a complementary method to assist other entity identification methods without redundant entity filtrations. Detailed surveys on biomedical text mining are made available in Cohen & Hersh [17], Zweigenbaum et al., [18] and Simpson and Demner-Fushman [19]. Extracted entities and entity relations can be further analyzed using techniques such as network centrality [20], statistical analysis [21], and citation analysis [22].

It is apparent from these studies that understanding various relations among biomedical entities is a cornerstone, because these entities are better understood by probing into their interactions with others. There is an emerging trend of applying bibliometric techniques to study biomedical entities, coined by the term “Entitymetrics” [23]. In Entitymetrics, entity-driven bibliometrics tackles the problems of knowledge transfer and discovery at three different levels: micro-, meso-, and macro-level. While many aforementioned studies mainly examined the ways of discovering biomedical entities and entity relations from scientific literature, there lacks an integrated research that uses extracted entities and entity relations to facilitate literature-based information discovery. Therefore, the goal of this study is to fill the gap between the techniques of entity and entity relation extraction and the application of these techniques to gain insights into scientific literature.

Specifically, the following two research questions will be investigated: 1) In biomedicine, given a body of scientific literature, what biomedical entities have a higher impact on others and thusly should be further studied? 2) Which pairs of entities have the potential to have meaningful relations for information discovery, entity and entity relation recommendation, and other retrieval and clinical applications? In this sense, our study servers as a bridge that connects prior studies on biomedical text mining with practical applications to assist more focused research through entities and their relations of the highest importance. To achieve this goal, we propose a framework for identifying important diseases, drugs, and genes for a given disease. The framework comprises an entity extraction method and a three-level network-based approach for the analysis of a literature-based dataset.

Cancer is a primary cause of death worldwide, among which, liver cancer is the second leading cause of cancer deaths [24]. As many as 564,000 people are diagnosed with liver cancer each year, and the trend tends to continue for several decades in several developed countries such as the United States [25]. It is known that most liver cancer instances started from other parts of the body and several types of tumors can grow in liver because liver comprises different types of cells [26]. Thus, in this broad scope of literature-based liver cancer study, identifying important entities and relations among entities that are highly relevant to liver cancer is seen as beneficial. In this regard, we apply the proposed methods to a publication dataset to understand the disease using this rich source of scientific literature.

## Data and Methods

### Data

“Liver cancer” was selected as the seed term to query PubMed. We retrieved 169,774 PubMed records and downloaded them in XML format. We then parsed the downloaded records to extract titles and abstracts for entity extraction by implementing a SAX Parsing module. Our dataset comprises 16,568 entities (S1 File. Entities) and 1,023,204 entity-entity and paper-entity relations (S2 File. Paper-Entity Relations). Table 1 shows the percentage of each type of entities among all 16,568 entities. The process of entity extraction from the downloaded records will be discussed in the method section.

**Table 1:** Entity types and their percentage.

Entity Type	Number	Percentage
Disease	9,681	58.44%
Drug	4,347	26.24%
Gene	2,540	15.32%

### Methods overview

**Fig 1:**
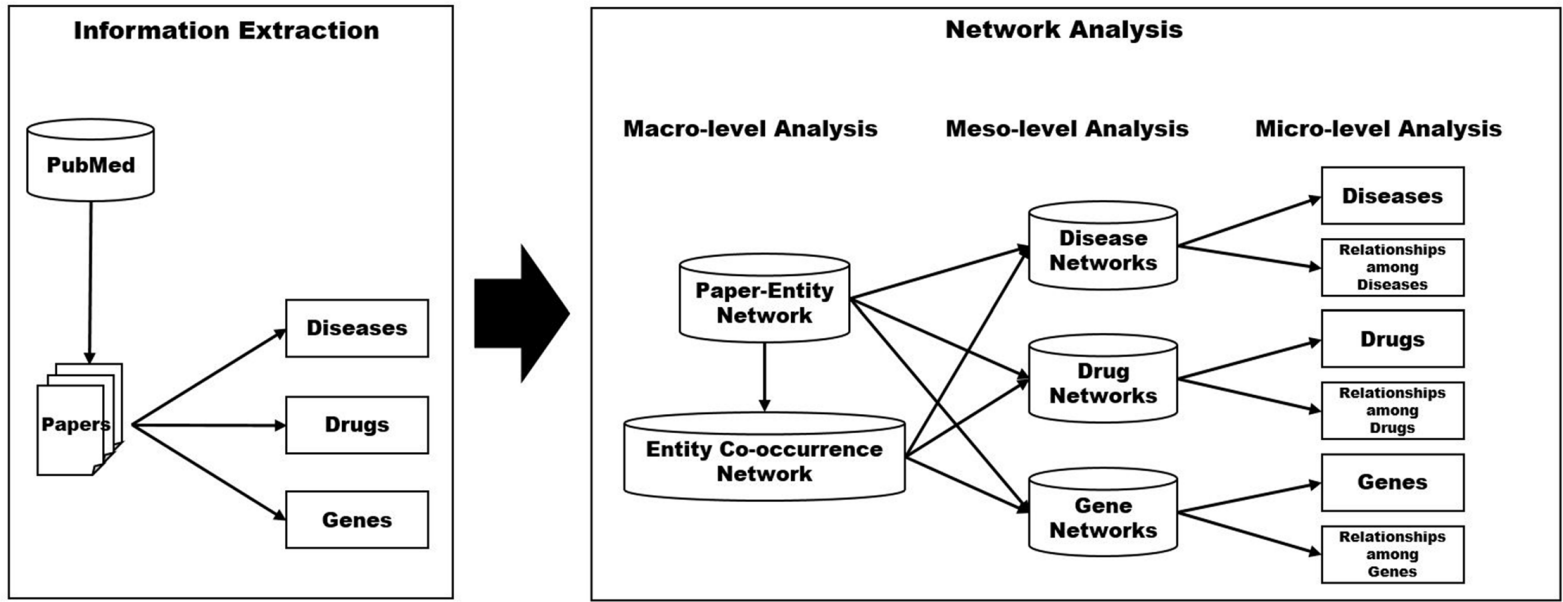
A schematic diagram for the proposed methods.

Disease, drug, and gene entities were extracted from articles obtained from PubMed. Extracted entities are used to construct a paper-entity network as well as an entity co-occurrence network. These macro-level networks were further decomposed into three types of meso-level networks (i.e., disease networks, drug networks, and gene networks). These entity specific networks are employed to investigate important diseases, drugs, and genes as well as salient relations within each entity group. Fig 1 shows the schematic diagram for the propose method.

We explain two main steps of the proposed method, information extraction and network analysis, in the following subsections.

#### Information Extraction

We implemented an entity extraction module by extending Stanford CoreNLP [27]. Stanford CoreNLP provides a set of natural language processing (NLP) analysis tools which can take English language texts and perform a variety of NLP tasks such as sentence splitting, Part-Of-Speech (POS) tagging, and dependency parsing. The entity extraction module went through the following four steps. The first step is to split a record into sentences. We used the “ssplit” pipe provided in Stanford CoreNLP. The second step is to build three dictionaries for diseases, genes, and drugs. We used the CTD database (http://www.ctdbase.org/) to create the three dictionaries. In total, the drug dictionary comprises 151,729 drug names; the disease dictionary comprises 11,937 disease names; and the gene dictionary comprises 297,514 gene names. The third step is to incorporate PubTator [28] to strengthen the inducted dictionaries. We conducted a preliminary test of extracting bio-entities only with CTD-based dictionaries and found that the quality of entity extraction was not satisfactory. Thus, we decided to add PubTator to further strengthen the dictionaries. PubTator, developed to fulfill two curation tasks—document triage and bio-concept annotation, contains bio-entity annotations for several entities such as chemicals, diseases, genes, mutations, and species. Out of these types, we are only interested in disease, drug, and gene types. Pubtator contains 16,582,474 genes, 26,788,622 diseases, and 24,915,999 drugs. When we merged three CTD dictionaries with three PubTator dictionaries for drug, disease, and gene, we checked if there is a common dictionary entry shared in both dictionaries. If found, we only kept one entry. This duplicate elimination step results in 25,053,123 drug names for the drug dictionary, 26,791,436 disease names for the disease dictionary, and 16,761,566 gene names for the gene dictionary. The fourth step is to match tokenized phrases to the three dictionaries. In this step, we employed the following three sub-steps: tokenization, lemmatization, and normalization.

Tokenization. We used the Stanford PTBTokenizer tokenization technique [29]. PTBTokenizer is designed to be a fast, rule-based tokenizer to conform to the Penn Treebank tokenization conventions [29].

Lemmatization. We used the lemmatization technique which is available in the Stanford CoreNLP package. It provides full morphological analysis for accurate identification of the lemma for each word. Lemmatization is similar to word stemming, but rather than produce a stem of the word, it replaces the suffix to get the normalized word form.

Normalization. We used the string normalization technique to reduce the string variation by case sensitivity and special characters including +, *,;, and _. Strings with uppercase are changed to those with lowercase, and/or the appointed special characters are removed from all the input texts and dictionary data. In the case of the special character ‘-’, it is replaced by whitespace, allowing for the general entity name patterns.

#### Network Analysis

**Fig 2:**
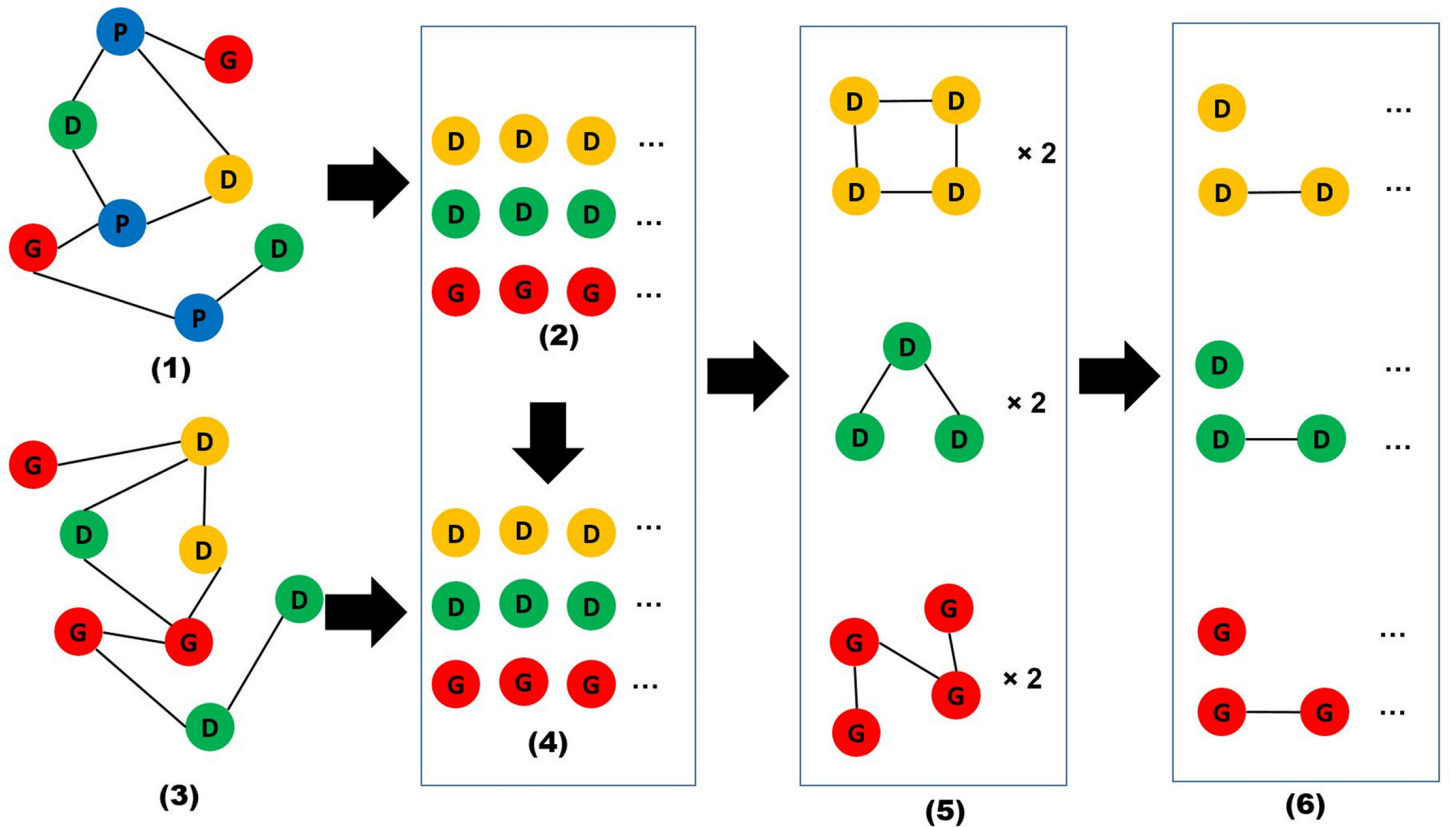
A flow chart of six steps.

The network analysis comprises six steps (Fig 2): 1) the construction of a paper-entity network; 2) the identification of top entities; 3) the construction of an entity co-occurrence network; 4) the identification of entities highly co-occurred with top entities; 5) the construction of entity specific networks (PageRank- and betweenness-based); and 6) the exploration of entity specific networks. These steps are elaborated in the following paragraphs.

A paper-entity network was constructed using the extracted entities. It is a heterogeneous, unweighted network that contains four types of nodes: papers, diseases, drugs, and genes. The network captures relations between papers and entities so that there is an edge if a paper includes an entity (i.e., a disease, drug, or gene). The paper-entity network forms the basis for identifying important entities through topological investigations. Two network-based measures, PageRank and betweenness centrality, were used to identify important entities from this network. PageRank is an algorithm used to rank web pages according to the impact of inlinks [30]. Entities ranked highly by PageRank are those with the highest impact. Betweenness centrality is an indicator of measuring the influence of nodes in terms of the ability to transfer information in a network [31]. Thus, a node with high betweenness centrality means it plays an important role in transferring information to others. In the paper-entity network, entities with a high betweenness centrality play a key role in the whole network by connecting other entities. These two algorithms have been applied to a number of areas to identify important artifacts and actors. For instance, Zhu & Yan [32] applied PageRank to identify important subfield within computer science to understand its knowledge diffusion patterns; Jing & Baluja [33] applied PageRank to retrieve highly relevant images in an image search. Likewise, betweenness centrality was employed to identify important nodes to solve the problem of network control in communication networks [34]; it was also applied to an alliance network to explore novel technologies [35].

A co-occurrence network was then constructed from the paper-entity network. The co-occurrence network is a heterogeneous, weighted network comprises diseases, drugs, and genes. Paper-entity relations were used to calculate co-occurrence values. That is, if two or more entities co-occurred within a paper, the number of co-occurrence was recorded and treated as the weight in the entity co-occurrence network. Co-occurrence networks have been widely studied [36, 37], based on the notion that entities have strong interactions with each other tend to co-occur frequently. Thus, co-occurrence relations are an important feature in examining between-entity relations.

In an entity co-occurrence network, diseases that highly co-occurred with top diseases identified from the paper-entity network were then extracted. Because we have two sets of top diseases identified separately from PageRank and betweenness centrality, two disease specific datasets were collected. Four more datasets (i.e., on drugs and genes) were also constructed separately by using the same method. Thus, each of the six datasets includes top entities and entities that highly co-occurred with these top entities. The six datasets were then used to construct six homogeneous networks (i.e., two disease networks (PageRank-based and betweenness centrality-based), two drug networks, and two gene networks) by reserving the co-occurrence value as link weight. These six networks are the transformed networks of the previous entity co-occurrence network by only including one type of entities as well as a small set of important entities. The entity specific networks are constructed to get a condensed and meaningful view of the seed disease. In each of the six entity networks, we also extracted highly co-occurred entity pairs. Because each entity type is associated with two entity specific networks (PageRank-based and betweenness centrality-based), two sets of pairs within an entity type were identified.

## Results

In this section, we sequentially report important diseases, drugs, and entities as well as important pairs of entities in the area of liver cancer research.

### Important Entities

Table 2 shows two sets of top 10 disease, drugs, and genes: one based on PageRank and the other based on betweenness. We discuss these important entities in the following three subsections.

**Table 2:** Top entities highly ranked by PageRank and Betweenness centrality.

Rank	Disease		Drug		Gene	
	PageRank	Betweenness	PageRank	Betweenness	PageRank	Betweenness
1	Tumor	hepatoma	alcohol	gamma-glutamyl	HCC	recombinant human interleukin 2
2	hepatocellular carcinoma	Tumor	cisplatin	Tyrosine	AFP	creatine kinase B
3	Cancer	Cancer	glucose	trastuzumab	p53	G21
4	HCC	autosomal recessive, inherited disorder	oxygen	metallocorrole	CEA	gamma-glutamyl transpeptidase
5	Hepatoma	intrahepatic and extrahepatic cholangiocarcinoma	tyrosine	glutamyl	albumin	ED2
6	Cirrhosis	CRLM and extra hepatic disease	ethanol	[11C]CH3OTf	TACE	CDH2
7	Hepatitis	Thyrotoxicosis	5-FU	3-methylcholanthrene	insulin	thyroid hormone receptor beta
8	colorectal cancer	mitochondrial dysfunction	bilirubin	calcium folinate	alpha-fetoprotein	vascular-endothelial growth factor and fibroblast growth factor receptors
9	liver metastasis	HPV	glutathione	CBD	VEGF	Histone
10	liver cirrhosis	absence of disease progression, fatty liver	amino acid	diethylnitrosamine	IL-6	beta-galactosidase

#### Diseases

As shown in Table 2, three diseases (i.e., tumor, cancer, and hepatoma) appeared in both lists. Hepatocellular carcinoma, HCC, and hepatoma denote the same concept and so do cirrhosis and liver cirrhosis. Hepatocellular carcinoma is one common type of liver cancer caused by cirrhosis in most cases. Cirrhosis/liver cirrhosis might be caused by hepatitis [38]. Compare to PageRank, betweenness centrality includes more specific terms (i.e., autosomal recessive, inherited disorder, intrahepatic and extrahepatic cholangiocarcinoma, and CRLM and extra hepatic disease) and terms that may not be easily associated with liver cancer such as thyrotoxicosis, mitochondrial dysfunction, and HPV. These diseases’ connections to liver cancer might be the ones that have the potential to be further understood.

#### Drugs

Unlike diseases, only one drug (i.e., tyrosine) appeared in both lists. Tyrosine or Tyrosine kinase inhibitor (TKI) is a drug used to treat liver cancer by inhibiting Tyrosine kinases which are enzymes used by cells to transmit growing and dividing signals [39, 40]. Trastuzumab is used to treat breast cancer and malignant tumors [41] and calcium folinate is used to reduce side-effects caused by using some anti-cancer medicines [42]. Betweenness centrality ranks chemical compounds highly such as metallocorrole, [11C]CH3OTf, 3-methylcholanthrene, CBD (Cannabidiol), and diethylnitrosamine. We give s brief overview to some important drugs in this section.

- Cisplatin: Cisplatin is used to treat various cancers including liver cancer [43].
- Glucose: Liver cells are known to produce glucose which helps human maintain healthy blood-sugar levels. If these cells become cancerous, then they lose the ability and this makes tumor cells proliferate [44].
- 5-FU: 5-fluorouracil is a drug used to treat cancer [45].
- Glutathione: Glutathione, also known as gamma-glutamyl, is a substance contained in cells. It is taken to detoxify and prevent heart diseases, various cancers, etc. [46].

Besides these drugs, some basic elements, such as oxygen, amino acid, tyrosine (one of the 22 amino acids) are also highly ranked by PageRank. These elements have the ability to stimulate body functions and repair body tissues.

#### Genes

Unlike diseases and drugs, two lists in Table 2 do not share any common gene. Because genes are more granular entities than diseases and drugs, they may not exclusively relate to liver cancer. Readers can visit GeneCards (http://www.genecards.org), a human gene database, for more information on these genes.

### Network Characteristics of Entity Networks

Top entities shown in Table 2 were used to identity other entities that highly co-occurred with these entities in entity co-occurrence networks. Then, these entities altogether form two disease networks (PageRank-based and betweenness centrality-based), two drug networks, and two gene networks, from which we identified top pairs of diseases, drugs, and genes. Table 3 shows the statistics of each network.

**Table 3:** Network characteristics for the six entity networks.

Indicators	Disease		Drug		Gene	
	PageRank	Betweenness	PageRank	Betweenness	PageRank	Betweenness
No. of Nodes	41	55	77	58	67	47
No. of Edges	79	64	93	63	86	40
Avg. Degree	3.85	2.33	2.42	2.17	2.57	1.7
Avg. Weighted Degree	2210	836	156	46	124	6
Avg. Path Length	2.79	3.34	3.43	3.2	3.39	3.06
Graph Density	0.096	0.043	0.032	0.038	0.039	0.037
Modularity	0.19	0.1	0.71	0.59	0.46	0.54
No. of Communities	2	7	7	8	6	10
Avg. Clustering Coefficient	0.11	0.44	0.49	0.51	0.48	0

As shown in Table 3, PageRank-based networks have higher average degrees as well as average weighted degrees. This indicates that entities in PageRank-based networks interact more actively with each other. For average path length, each network has a similar average path length (i.e., about 3). All networks are sparse with graph density lower than 0.1. Modularity is used to measure the likelihood that a network can be divided into communities [47]. Disease networks have lower modularity than the drug and gene networks. This is because diseases generally interact with many other diseases. While betweenness centrality-based networks have more communities than PageRank-based networks, the PageRank-based disease network only has two communities, which is much lower than the minimum number of communities of other networks. Betweenness centrality-based gene network recorded an average clustering coefficient of zero. This suggests that there is no triangle in this network, as genes shown in Table 2 (betweenness centrality-based) have rather distinct characteristics.

### Salient Pairs of Diseases, Drugs, and Genes

Table 4 shows highly co-occurred pairs of diseases, drugs, and genes. These pairs were divided into three groups based on the number of co-occurrence. We discuss these important entity pairs in the following three subsections.

**Table 4:** Top 15 entity pairs.

Range	Rank	Disease		Drug		Gene	
		PageRank	Betweenness	PageRank	Betweenness	PageRank	Betweenness
> = 1000	1	tumor—hepatocellular carcinoma	tumor—hepatocellular carcinoma	**cisplatin—doxorubicin**	N/A	HCC—AFP	N/A
> = 1000	2	tumor—HCC	tumor—HCC	bilirubin—aspartate	N/A	HCC—TACE	N/A
> = 1000	3	tumor—liver metastasis	tumor—liver metastasis	**cisplatin—5-fluorouracil**	N/A	HCC—p53	N/A
> = 1000	4	cirrhosis—hepatitis	tumor—hepatoma	**alcohol—ethanol**	N/A	HCC—VEGF	N/A
> = 1000	5	hepatocellular carcinoma—liver cirrhosis	cancer—hepatocellular carcinoma	**oxygen—superoxide**	N/A	HCC—alpha-fetoprotein	N/A
<1000 and > = 100	1	hepatitis—chronic hepatitis	cancer–HCC	**cisplatin—platinum**	diethylnitrosamine—phenobarbital	AFP—DCP	N/A
<1000 and > = 100	2	cancer—HCC	tumor—metastasis	tyrosine—serine	tyrosine—serine	**p53—Bcl-2**	N/A
<1000 and > = 100	3	HCC—liver cirrhosis	cancer—liver metastasis	**5-FU—folinic acid**	3-methylcholanthrene—phenobarbital	**p53—p21**	N/A
<1000 and > = 100	4	HCC—chronic hepatitis	tumor—liver cirrhosis	**cisplatin—adriamycin**	tyrosine—sorafenib	**insulin—glucagon**	N/A
<1000 and > = 100	5	tumor—metastasis	cancer—colorectal cancer	**cisplatin—CDDP**	**gamma-glutamyl—glutamyl**	HCC—IFN	N/A
<100	1	cirrhosis—viral hepatitis	hepatoma—hepatitis B	**5-FU—uracil**	**tyrosine—imatinib**	TACE—RFA	gamma-glutamyl transpeptidase—alkaline phosphatase
<100	2	hepatoma—hepatitis B	hepatoma—liver cirrhosis	**alcohol—aldehyde**	tyrosine—threonine	IL-6—IL-10	gamma-glutamyl transpeptidase—HCC
<100	3	liver cirrhosis—liver cancer	hepatoma—liver tumor	**glucose—phosphoenolpyruvate**	diethylnitrosamine—glutathione	VEGF—CD34	gamma-glutamyl transpeptidase—AST
<100	4	liver metastasis—liver tumor	hepatoma—breast cancer	**tyrosine—imatinib**	diethylnitrosamine—2-acetylaminofluorene	**p53—Bax**	histone—HCC
<100	5	cirrhosis—hepatitis C	hepatoma—chronic hepatitis	**ethanol—PEIT**	diethylnitrosamine—glucose	IL-6—TNF-alpha	**histone—HDAC**

#### Diseases

**Fig 3:**
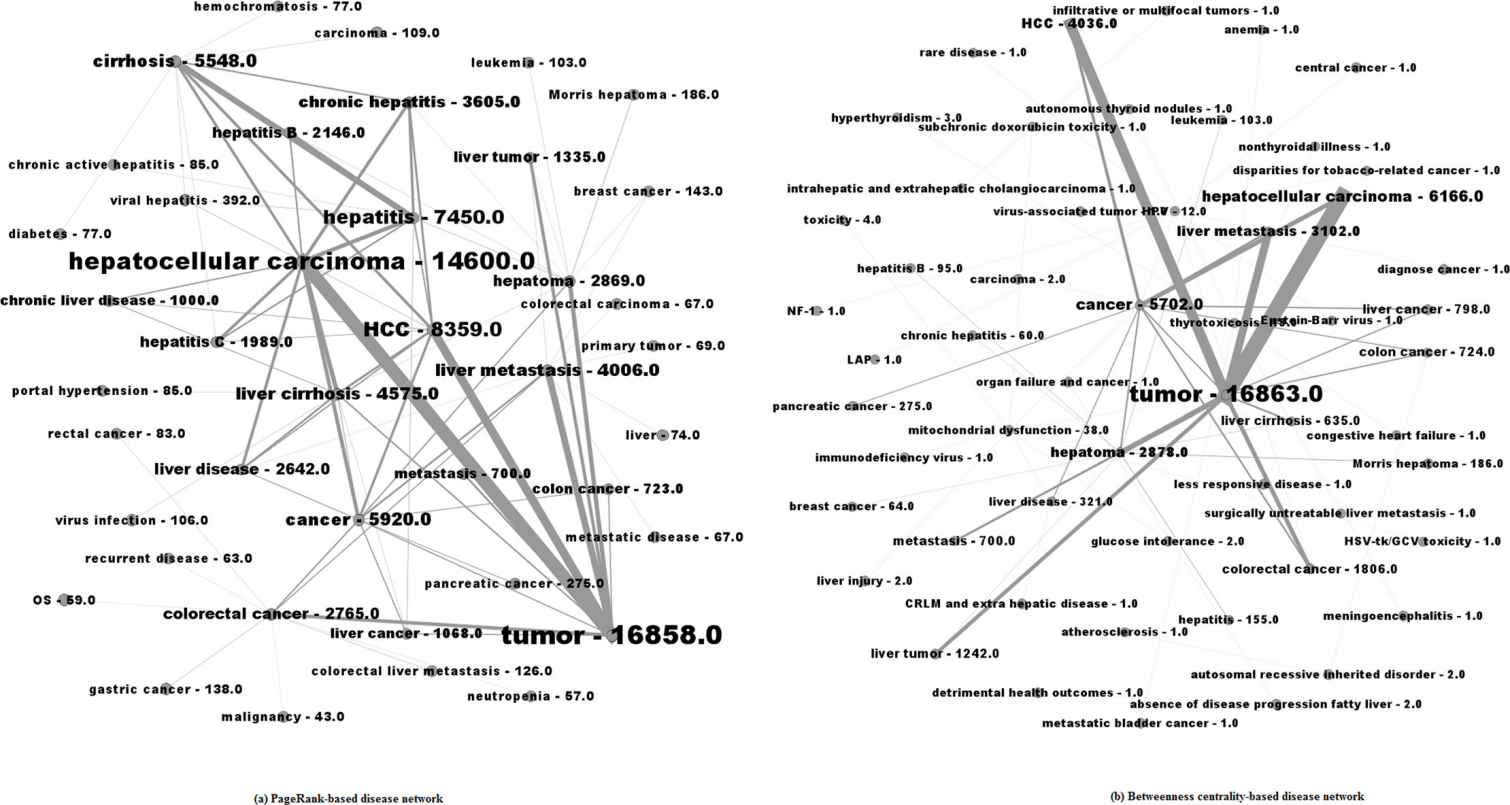
PageRank-based (a) and Betweenness centrality-based (b) disease networks.

PageRank-based and betweenness centrality-based disease networks are visualized in Fig 3. Node labels are proportional to weighted degree and the width of links are proportional to the number of co-occurrence between two diseases.

The most important entity in Fig 3(A) is tumor. Tumor highly co-occurred with hepatocellular carcinoma, HCC, cancer, and hepatoma. Important diseases in Fig 3(A) are generally the same diseases that are highly ranked by PageRank in Table 2.

Diseases in Fig 3(B) tend to co-occur infrequently with each other, which is in contrast with the PageRank-based disease network. One possible explanation is that top diseases with high betweenness centrality were not studied much in papers; thus, they did not co-occur frequently with other diseases.

Six pairs of diseases (i.e., tumor—hepatocellular carcinoma, tumor–HCC, tumor—liver metastasis, cancer–HCC, tumor–metastasis, and hepatoma—hepatitis B) appeared in both lists. Relations of these diseases are self-explanatory, probably with the exception of “hepatoma-breast cancer”. Recent discoveries have found that breast cancer, similar to cancers such as colon cancer, bladder cancer, and kidney cancer, is one of the cancers that may spread to livers [48].

#### Drugs

**Fig 4:**
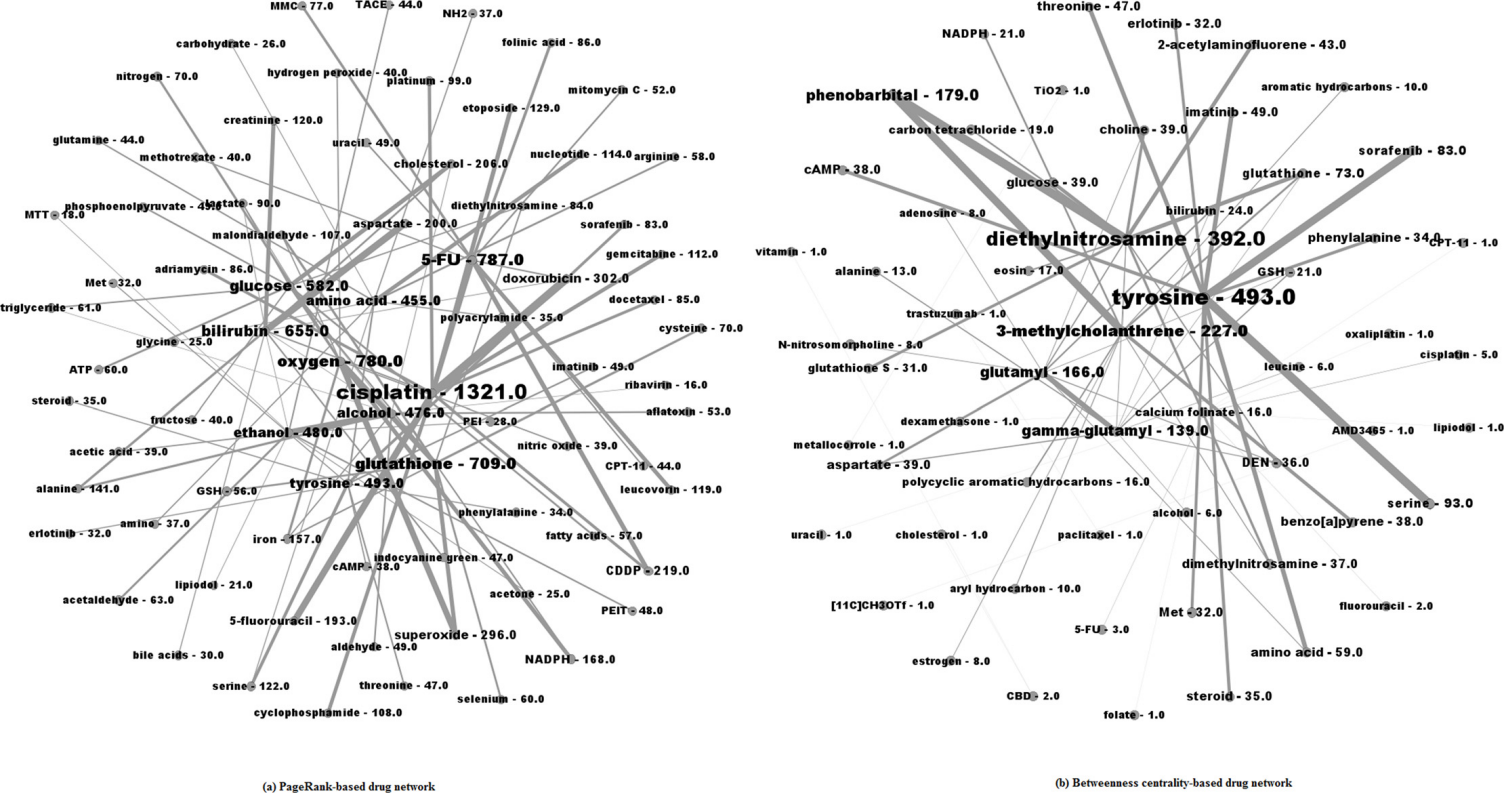
PageRank-based (a) and Betweenness centrality-based (b) drug networks.

Fig 4 shows two types of drug networks constructed from the paper-entity network and the entity co-occurrence network.

Ten most visible entities shown in Fig 4(A) are exactly the same as top 10 entities ranked by PageRank in Table 2 while the level of visibility is different.

Two important drugs in Fig 4(B) are tyrosine and diethylnitrosamine. Tyrosine, as mentioned in the previous section, is used to treat liver cancer by inhibiting Tyrosine kinases [36]. Diethylnitrosamine, ranked the second, co-occurred 392 times with other drugs. The status of diethylnitrosamine is more apparent in the drug specific network (ranked the second) than in the paper-entity network (ranked the 10th). This finding has supported the need to construct such entity specific networks―by doing so, we are able to gain more granular understanding of the interactivity of entities which may be overlooked in the global network.

Top 15 drug pairs are shown in Table 4. Two pairs (i.e., tyrosine–serine and tyrosine–imatinib) are shown in both PageRank- and betweenness centrality-based lists. Both tyrosine and serine belong to the same group- proteingenic amino acids which are building blocks of proteins [49]. Imatinib is one kind of tyrpsine-kinase inhibitor used for treating cancers. In the list of betweenness centrality, there is no pair occurred more than 100 times.

Relations shown in Table 4 were examined by referencing online resources including WebMD (http://www.webmd.com) and Drugs.com (http://www.drugs.com). These websites provide detailed information on drugs as well as drug interaction checker services. Relations that were mentioned by the two online resources were bold-faced in Table 4. Only two relations (i.e., bilirubin-aspartate and tyrosine-serine) were not confirmed in PageRank-based list while in the betweenness centrality-based list, two relations (i.e., gamma-glutamyl–glutamyl and tyrosine–imatinib) were confirmed. Thus, literature-based approach is a valuable way to assist clinical trials.

#### Genes

**Fig 5:**
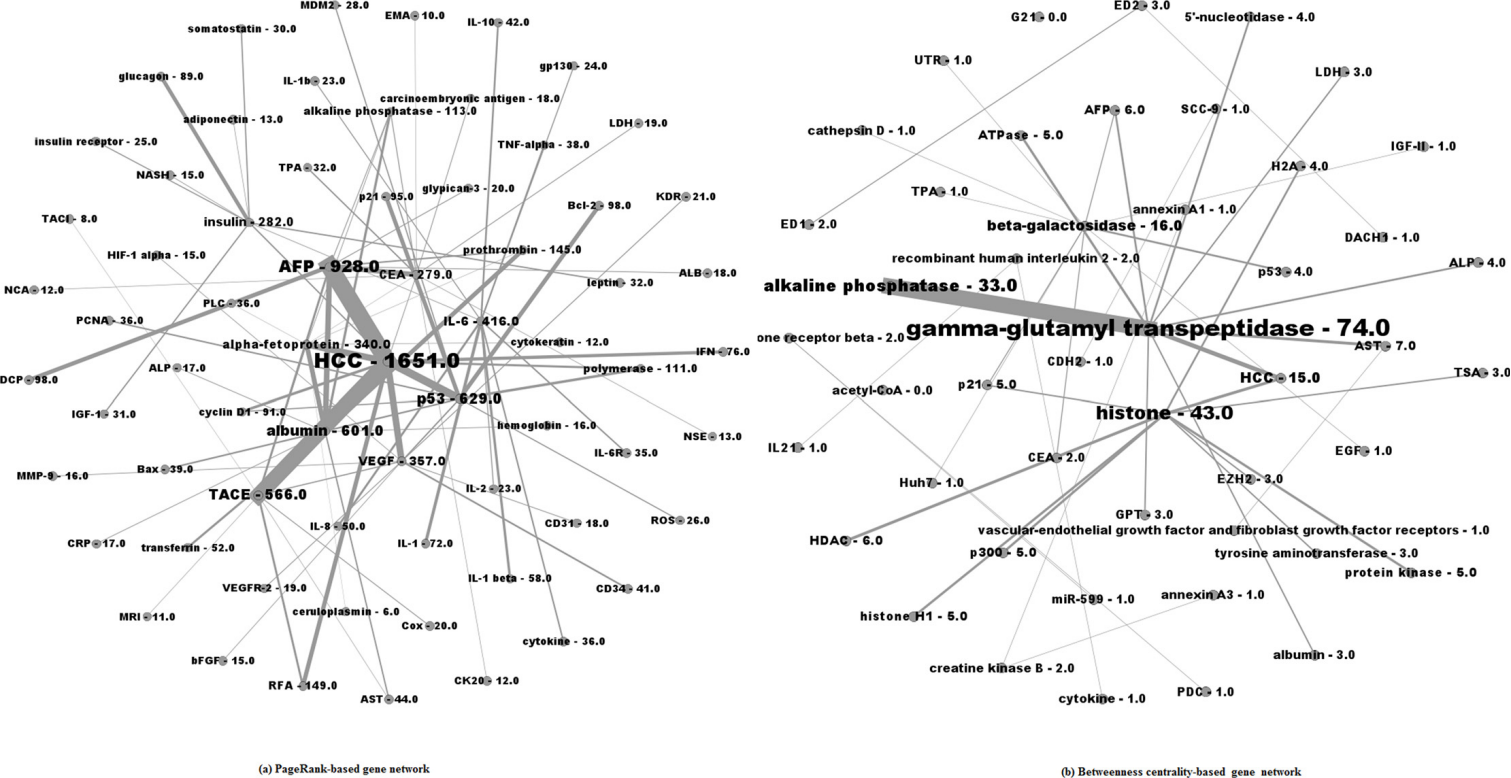
PageRank-based (a) and Betweenness centrality-based (b) gene networks.

Fig 5 illustrates two gene specific networks constructed from a collection of top genes and genes that highly co-occurred with these genes.

Betweenness centrality-based gene network includes 47 significant genes, which has fewer genes than the PageRank-based one that has 67 genes. One feature of Fig 5(B) is that most genes co-occurred less than five times with other genes. This suggests that they were not widely studied in previous literature and interactions between these genes and liver cancer may need to be further investigated.

Table 4 shows top 15 gene pairs identified from the PageRank- and betweenness centrality-based gene network. All pairs in the list of betweenness centrality occurred less than 50 times. Investigating interactions between diseases and genes may be more difficult than looking into the relations between diseases and diseases/drugs because genes are more granular entities and may actively or latently relate to a slew of diseases or drugs. In this sense, interactions shown in this study can be used to initiate a meaningful research.

To examine gene relations in Table 4, we referenced online resources including BioGRID (http://www.thebiogrid.org), BioGraph (http://www.biograph.be), CTD (http://www.ctdbase.org), and GeneCards (http://www.genecards.org). BioGRID confirmed three relations (i.e., p53-Bcl-2, p53-Bax, and histone-HDAC), BioGraph confirmed one relation (i.e., p53-p21), and CTD confirmed one relation (i.e., insulin-glucagon). Relations that were confirmed by these online resources were bold-faced in Table 4. Unlike diseases and drugs, a number of gene relations in Table 4 were not confirmed by clinical trials. This is probably due to the large volume of genes and their relations that may relate to liver cancer.

## Discussion and Conclusions

In this study, we proposed a literature-based approach for identifying disease-related entities that include diseases, drugs, and genes for liver cancer. A series of network-based approaches were applied to identify important entities among the extracted entities. Top diseases, drugs, and genes were identified by two distinct measures and thusly two groups of entities were obtained. One group, formed based on entities that have the highest PageRank scores, includes entities that gained popularity and were widely investigated in literature. Entities included in this group are important in understanding diseases. The other group, formed based on entities that have the highest betweenness centrality, includes entities that played key roles in the whole network in connecting other entities. Entities in this group possibly possess topological importance in studying the given disease. Six entity specific networks were constructed by combining the entity co-occurrence network and the identified top entities to discover salient entity relations. A portion of the discovered entity relations was verified by clinical trials.

Key findings were obtained: 1) PageRank and betweenness centrality are complementary in identifying important entities. As PageRank identifies popular entities while betweenness centrality identifies influential entities, the combinatorial use of the two is a reasonable and effective way to select and examine important entities; 2) the integrative use of global and regional networks effectively identifies global entities as well as entities that are important, but not noticeable in the global topology. Regional networks make it possible to identify important pairs of entities from a large volume of links in global networks; 3) diseases, drugs, and genes present different characteristics in identifying important entities and pairs of entities that relate to liver cancer. Identified diseases and pairs of diseases have the highest familiarity while the interpretation of identified drugs and genes imposes more challenges as shown in the cross-validation of the results with external resources. This implies an increased level of demandingness in bio-entity research as the studied entities become more granular. Thus, similar research in a more detailed level is promising and critical in advancing literature-based biomedical research; and 4) some relations identified by the proposed method have a high consistency with clinical trials (i.e., drug relations) while some does not (i.e., gene relations). Unconfirmed relations do not mean unimportant relations; rather, they stand out among many others because they signify potentially important relations that might be validated in future research. Researchers and practitioners can take the results of literature-based approach as an initiating point of their research. The proposed method can serve to assist clinical trials to identify important entity relations.

This study has some limitations. Links among entities were based on co-occurrence relations. Co-occurrence may not directly demonstrate actual interactions among entities. Because the goal of this study is to propose a supplementary way to discover key biomedical information, the proposed methods should be of value to assist biomedical studies, particularly from the aspect of identifying latent biomedical entities and their relations. Due to the exploratory nature of the study, results have included both known facts that have been proven by others and also hypotheses that need to be verified in future studies. This means there is no standard guideline and test dataset to compare the performance of our approach. Instead, the identification results were verified by consulting facts published in scientific articles and authoritative biomedical resources.

As there will be more scientific literature available in the future, automated knowledge mining from text will become vital. The proposed method is an applicable and effective way to achieve this goal by integrating a series of information extraction and network-based approaches. Future studies may need to focus on other relations among entities by applying advanced statistical and natural language processing techniques, such as side effects, induce, and inhibit relations. As the infrastructures and techniques of processing big data are improving, the discovery of latent knowledge will be accelerated by applying the proposed literature-based approaches to a large volume of scientific literature—we see this as another valuable thread of research.

## Supporting Information

S1 File Entities. (ZIP)

S2 File Paper-Entity Relations. (ZIP)
